# Measures of Financial Hardship From Health Care Expenses Among Families With a Member With Atherosclerotic Cardiovascular Disease in the US

**DOI:** 10.1001/jamahealthforum.2022.1962

**Published:** 2022-07-22

**Authors:** Stephen Y. Wang, Javier Valero-Elizondo, Miguel Cainzos-Achirica, Nihar R. Desai, Khurram Nasir, Rohan Khera

**Affiliations:** 1 Department of Internal Medicine, Yale New Haven Hospital, New Haven, Connecticut; 2 Center for Outcomes Research, Houston Methodist, Houston, Texas; 3 Division of Cardiovascular Prevention and Wellness, Department of Cardiology, DeBakey Heart & Vascular Center, Houston Methodist, Houston, Texas; 4 Section of Cardiovascular Medicine, Department of Internal Medicine, Yale School of Medicine, New Haven, Connecticut; 5 Center for Outcomes Research and Evaluation, Yale New Haven Hospital, New Haven, Connecticut

## Abstract

**Importance:**

Patients with atherosclerotic cardiovascular disease (ASCVD) face substantial financial burden from health care costs as assessed by many disparate measures. However, evaluation of the concordance of existing measures and the prevalence of financial burden based on these measures is lacking.

**Objective:**

To compare subjectively reported and objectively measured financial burden from health care in families of patients with ASCVD.

**Design, Setting, and Participants:**

This cross-sectional study used data from the Medical Expenditure Panel Survey, a nationally representative survey of individuals and families in the US, and included all families with 1 or more members with ASCVD from 2014 to 2018. Analyses were conducted from October 2021 to April 2022.

**Main Outcomes and Measures:**

Using accepted definitions, objective financial hardship represented annual out-of-pocket medical expenses exceeding 20% of annual postsubsistence income, and subjective financial hardship represented self-reported problems paying medical bills or paying them over time. Prevalence of financial hardship was identified based on individual definitions and their concordance was assessed. Factors associated with each type of financial hardship were examined using risk-adjusted survey logistic regression. Multivariable logistic regression was used to model the odds of subjective financial hardship vs objective financial hardship across subgroups. The association between measures of financial hardship and self-reported deferral of care was also assessed.

**Results:**

Among 10 975 families of patients with ASCVD, representing 22.5 million families nationally (mean [SD] age of index individual, 66 [24] years; estimated 54% men]), 37% experienced either objective or subjective financial hardship. This group included 11% (95% CI, 10%-11%) with objective financial hardship, 21% (95% CI, 20%-22%) with subjective financial hardship, and 5% (95% CI, 5%-6%) with both objective and subjective financial hardship. Mean age was 70 (95% CI, 68-71) years vs 61 (95% CI, 60-62) years for index patients in families reporting objective financial hardship only vs subjective financial hardship only, with no difference in sex (50% [95% CI, 46%-54%] of men vs 49% [95% CI, 47%-52%] of women). In risk-adjusted analyses, among families of patients with ASCVD, patient age of 65 years or older was associated with lower odds of subjective financial hardship than objective financial hardship (odds ratio [OR], 0.39; 95% CI, 0.20-0.76), whereas higher income (OR, 6.08; 95% CI, 3.93-9.42 for an income of >100%-200% of the federal poverty level [FPL] vs ≤100% of the FPL and OR, 20.46; 95% CI, 11.45-36.56 for >200% of FPL vs ≤100% of FPL), public insurance (OR, 6.60; 95% CI, 4.20-10.37), and being uninsured (OR, 5.36; 95% CI, 2.61-10.98) were associated with higher odds of subjective financial hardship than objective financial hardship. Subjective financial hardship alone was associated with significantly higher adjusted odds of self-reporting deferred or forgone care compared with objective financial hardship alone (OR, 2.69; 95% CI, 1.79-4.06).

**Conclusions and Relevance:**

In this cross-sectional study of US adults, 2 in 5 families of patients with ASCVD experienced health care–related financial hardship, but a focus on objective or subjective measures alone would have captured only half the burden and not identified those deferring health care. The findings suggest that a comprehensive framework that evaluates both objective and subjective measures is essential to monitor financial consequences of health care.

## Key Points

**Question:**

Is there concordance between subjective and objective measures of financial burden in families of patients with atherosclerotic cardiovascular disease (ASCVD)?

**Findings:**

In this cross-sectional study of 10 975 US families, 2 in 5 families of patients with ASCVD experienced health care–related financial hardship; a focus on objective or subjective measures alone would have captured only half the total financial burden and not identified those forgoing or deferring health care.

**Meaning:**

The findings suggest that a comprehensive framework that evaluates both subjective and objective measures is needed to monitor financial consequences of health care in families of patients with ASCVD.

## Teaser

This cross-sectional study compares the financial burden of atherosclerotic cardiovascular disease among families of patients with objective or subjective financial hardship.

## Introduction

Financial challenges from health care costs pose a considerable burden on patients with atherosclerotic cardiovascular disease (ASCVD) in the US. Almost half of adults with ASCVD self-report problems paying medical bills.^1^ Furthermore, 1 in 7 families with ASCVD experience large annual health-related out-of-pocket expenses relative to their income, with most associated with health insurance premiums or medication costs.^2^

Given the high out-of-pocket cost of health care for many patients in the US, it is essential to evaluate common measures of financial burden from health care to guide policy efforts. Previous literature on financial hardship, mostly in oncology, has considered the following 2 widely used approaches to gauging financial burden: subjective financial hardship ascertained by self-report of difficulty paying medical bills^1^ and objective financial hardship, which is defined as annual out-of-pocket medical costs greater than 20% of annual postsubsistence income.^2^ Although the terms *subjective financial hardship* and *objective financial hardship* are often used interchangeably as measures of financial burden, they conceptually measure different aspects of the challenges patients may experience because of health care expenses. *Subjective financial hardship* and *objective financial hardship* have not been used concurrently in published reports on cardiovascular diseases. Even in the oncology literature, the prevalence of the terms has been qualitatively assessed in systematic reviews of published reports^3,4^ without broad concurrent assessments. However, whether objective and subjective measures of financial hardship are good surrogates of one another among individuals remains to be elucidated.

In a large national data set of community-dwelling individuals that captured both objective and subjective measures of financial burden in health care, we sought to evaluate commonly used measures of health care–related financial burden on families of patients with ASCVD. The aim of the study was to assess the concordance between objective and subjective measures of financial burden for patients and their families.

## Methods

### Data Source

This cross-sectional study used data from 2014 to 2018 from the Medical Expenditure Panel Survey (MEPS),^5^ an annual survey funded by the Agency for Healthcare Research and Quality. The study used calendar-year consolidated data sets from the Household Component of MEPS, which included cross-sectional information from a nationally representative sample of individuals and families in the US. The Household Component of MEPS was completed using a panel form, complex cross-sectional survey design, surveying individuals and families 5 times over a 2-year period, with oversampling of Asian, Black, and Hispanic individuals. The individuals surveyed were based on the respondents of the previous year’s National Health Interview Survey, and response rates approached 50%.^6^ Sampling weights were based on the relative proportion of families sampled and the national proportions of the corresponding family units.^7^ MEPS is reviewed and approved annually by the Westat Institutional Review Board.^8^ Because the study used fully deidentified publicly available data representing non–human participant research under 45 Code of Federal Regulations, §46 Protection of Human Subjects, it was outside the purview of the Yale University Institutional Review Board. The study followed the Strengthening the Reporting of Observational Studies in Epidemiology (STROBE) reporting guideline.

### Study Population

All families with an adult aged 18 years or older with ASCVD were included in the analysis. Atherosclerotic cardiovascular disease was identified from patient self-report as ever having been told by a doctor if the patient had coronary heart disease, angina, a heart attack, or stroke^2^ and diagnosed using the *International Classification of Diseases, Ninth Revision* and *International Statistical Classification of Diseases and Related Health Problems, Tenth Revision* codes.^7^ Similar to previous studies, we used families as the primary unit of study^2,9^ because cost burden is often shared among family members. Families were defined as 1 or more people related by birth, marriage, or adoption who were living together. If individuals were unrelated but lived in the same household, they represented separate families.^10^

### Study Covariates

We identified key sociodemographic variables of the study population, including age, sex, race and ethnicity (classified as self-reported Hispanic, non-Hispanic Black, non-Hispanic White, and other race and ethnicity, which included Asian, American Indian, and individuals with more than 1 race and ethnicity), income level, educational level (<high school diploma; high school diploma, General Educational Development and equivalent; or some college or higher degree), health insurance status (private, public, or uninsured), census region (Northeast, Midwest, South, and West), family size, and number of family members with ASCVD.

Total annual household income was used for this study, which was a composite of most types of taxable and nontaxable income, including wages, pensions, social security, and veteran payments, among others.^11^ Income categories included 100% or less of the federal poverty level (FPL), more than 100% to 200% of the FPL, and more than 200% of the FPL.^12,13^ An individual’s insurance type was reported monthly, and if an individual had both private and public insurance in a given year, their insurance type was identified as whichever type they used for a greater number of months in the year or as private if they had both types of insurance for an equal amount of time. If a patient did not have health insurance for even 1 month in a year, they were deemed as uninsured. Public insurance included Medicare, Medicaid, TRICARE, or other state-funded public insurance.^10^

Medical comorbidities were defined based on *International Classification of Diseases, Ninth Revision* and *International Statistical Classification of Diseases and Related Health Problems, Tenth Revision* codes. The comorbidities included arthritis, cancer, asthma, chronic obstructive pulmonary disease, hepatitis, and chronic kidney disease (eTable 1 in the Supplement). Cardiovascular disease risk factors included hypertension, diabetes, dyslipidemia, current smoking status, obesity, and insufficient physical activity (defined as less than moderate physical activity 5 times per week).

### Study Outcomes

We used 2 outcomes of financial hardship—subjective financial hardship and objective financial hardship—and we evaluated a third outcome that assessed self-reported deferral of care. Subjective financial hardship was considered present when an affirmative response was given for individually directed questions about problems paying medical bills in the preceding 12 months or having medical bills being paid over time. At a family level, a family was considered as having subjective financial hardship if any of its members had an affirmative response for survey questions about these 2 considerations.^1^

Objective financial hardship was considered present when a family spent 20% or more of their annual postsubsistence income as out-of-pocket medical costs.^2,9^ Out-of-pocket medical costs were obtained and summed together at the family level. The spending categories included hospitalizations, outpatient visits, medications, emergency department visits, health insurance premium costs, and other out-of-pocket health care costs, including medical equipment. These out-of-pocket costs were identified based on participant interviews, and participants were encouraged to refer to billing documents if they were available.^10^ All costs were inflation adjusted to the year 2018.^14^ If a family had health care expenses with a postsubsistence income of $0 or less, they were also defined as having objective financial hardship. Postsubsistence income was calculated based on nomograms of food cost from the US Bureau of Labor Statistics.^15^

We also used an outcome of deferred or forgone care, which was included to evaluate associated behavior of those experiencing subjective financial hardship or objective financial hardship or both. A family was defined as having deferred or forgone care if any of its members reported either a delay in getting medical care, prescription medications, or dental care or an inability to get necessary medical care, prescription medications, or dental care. Because the survey questions for deferred or forgone care changed in 2018, this outcome encompassed only the years 2014 to 2017.

### Statistical Analysis

We categorized families into 4 mutually exclusive financial burden categories: no financial hardship, objective financial hardship only, subjective financial hardship only, or both objective financial hardship and subjective financial hardship. First, we described sociodemographic variables, risk factors, and comorbidities by the type of financial burden using survey linear regression to compare differences between means for continuous variables and Rao-Scott χ^2^ to compare differences between categorical variables. Second, we compared the prevalence of each financial burden category by subgroups of age, sex, race and ethnicity, 2 or more comorbidities, income level, and insurance status. Third, we used multivariable logistic regression to model the odds of subjective financial hardship vs objective financial hardship across subgroups of age, sex, race and ethnicity, income level, insurance status, educational level, region, number of comorbidities, family size, number of family members with ASCVD, cardiovascular disease risk factors (obesity, exercise, dyslipidemia, hypertension, diabetes, current smoking status), and individual comorbidities (arthritis, cancer, asthma, chronic obstructive pulmonary disease, hepatitis, chronic kidney disease). We also evaluated risk-adjusted odds of subjective financial hardship and objective financial hardship individually against both objective financial hardship and subjective financial hardship across the same subgroups, accounting for the same covariates. In addition, we compared odds of deferred or forgone care by type of financial burden, adjusting for risk factors and comorbidities.

We conducted sensitivity analyses for which those families without a positive postsubsistence income (ie, income higher than $0 after accounting for food-related expenses) were excluded from the definition for objective financial hardship. All analyses accounted for the complex, multistage probability survey design of MEPS, and we used family-level sampling weights to compute nationally representative estimates. Stata/SE, version 14 (StataCorp LLC) was used for the analyses. A 2-sided *P* < .05 was deemed to be statistically significant. For descriptive analyses, the index individual for comparisons was defined as the participant with ASCVD within the family. If a family had more than 1 member with ASCVD, the oldest individual represented the index patient in the analysis. Analyses were conducted from October 2021 to April 2022.

## Results

### Financial Hardship in Atherosclerotic Cardiovascular Disease

A total of 10 975 families had at least 1 member with ASCVD, representing 22.5 million families nationally (95% CI, 21.5-23.4 million families). Overall, index members with ASCVD had a mean (SD) age of 66 (24) years; an estimated 12.0 million (54%) were men, 10 million (46%) were women, 2.3 million (10%) were Hispanic, 2.6 million (12%) were non-Hispanic Black, 16.0 million (72%) were non-Hispanic White individuals, and 1.4 million (6%) individuals were other races and ethnicities (including Asian, American Indian, or more than 1 race and ethnicity). Of all families with a member with ASCVD, 37% had either objective financial hardship or subjective financial hardship. This group comprised 11% (95% CI, 10%-11%) who had objective financial hardship alone, 21% (95% CI, 20%-22%) who had subjective financial hardship alone, and 5% (95% CI, 5%-6%) who had both objective and subjective financial hardship. The prevalence of subjective financial hardship alone vs objective financial hardship alone was higher in younger individuals (mean [95% CI] age, 61 [95% CI, 60-62] vs 70 [95% CI, 68-71] years; *P* < .001), non-Hispanic Black individuals (15%; 95% CI, 13%-17% vs 11%; 95% CI, 9%-13%; *P* < .001), those with diabetes (37%; 95% CI, 34%-39% vs 33%; 95% CI, 29%-36%; *P* < .001), current smokers (25%; 95% CI, 22%-27% vs 12%; 95% CI, 9%-15%; *P* < .001), and individuals with obesity (42%; 95% CI, 39%-45% vs 33%; 95% CI, 29%-37%; *P* < .001). There was no difference in prevalence on the basis of sex (49% [95% CI, 47%-52%] vs 50% [95% CI, 46%-54%] in women). The prevalence of subjective financial hardship alone vs objective financial hardship alone was lower among non-Hispanic White individuals (69%; 95% CI, 66%-72% vs 75%; 95% CI, 72%-78%; *P* < .001); households with income of less than or equal to 100% to 200% of the FPL (11%; 95% CI, 10%-13% vs 41%; 95% CI, 36%-45%; *P* < .001), individuals with private insurance (47%; 95% CI, 44%-50% vs 61%; 95% CI, 57%-65%; *P* < .001), and individuals with cancer (23%; 95% CI, 21%-25% vs 31%; 95% CI, 27%-35%; *P* < .001) (Table 1).

**Table 1.** Characteristics of Families With One or More Members With ASCVD, by Financial Burden Categories

Characteristics	% (95% CI)				*P* value
	No financial hardship	OFH only	SFH only	Both OFH and SFH	
Unweighted sample, No.	6740	1190	2420	625	NA
Individuals in weighted sample, weighted No. (%)	14 128 984 (63)	2 370 769 (11)	4 811 783 (21)	1 167 404 (5)	NA
Age, y					
Mean (95% CI)	67 (67-68)	70 (68-71)	61 (60-62)	63 (61-65)	<.001
18-44	7 (6-8)	7 (5-8)	12 (10-14)	13 (9-18)	<.001
45-64	30 (28-32)	25 (22-29)	46 (43-48)	41 (36-46)	
≥65	63 (61-65)	68 (65-72)	42 (40-45)	47 (41-52)	
Sex					
Male	56 (54-58)	50 (46-54)	51 (48-54)	46 (41-51)	<.001
Female	44 (42-58)	50 (46-54)	49 (47-52)	54 (49-59)	
Race and ethnicity					
Hispanic	10 (9-12)	8 (7-10)	10 (8-12)	9 (7-12)	<.001
Non-Hispanic Black	10 (9-12)	11 (9-13)	15 (13-17)	13 (11-16)	
Non-Hispanic White	73 (71-75)	75 (72-78)	69 (66-72)	72 (68-76)	
Other^a^	6 (5-7)	6 (4-7)	6 (5-8)	5 (4-8)	
Income level					
≤100% of FPL	11 (10-13)	41 (36-45)	11 (10-13)	38 (34-43)	<.001
>100%-200% of FPL	19 (18-21)	29 (26-33)	27 (24-29)	34 (29-38)	
>200% of FPL	69 (67-71)	30 (26-34)	62 (60-65)	28 (23-33)	
Educational level					
<High school diploma	12 (10-13)	13 (11-16)	12 (11-14)	13 (10-17)	.03
High school diploma, GED, and equivalent	38 (36-40)	43 (39-47)	42 (39-45)	39 (34-45)	
Some college or higher degree	50 (49-52)	44 (40-48)	46 (43-49)	48 (42-54)	
Insurance status					
Private	54 (52-56)	61 (57-65)	47 (44-50)	48 (44-53)	<.001
Public	43 (41-45)	36 (32-40)	47 (44-50)	43 (38-48)	
Uninsured	3 (3-4)	3 (2-4)	6 (5-7)	9 (6-12)	
Census region					
Northeast	18 (16-20)	18 (15-22)	16 (13-18)	14 (11-17)	<.001
Midwest	22 (20-24)	22 (19-25)	25 (22-28)	23 (22-28)	
South	39 (37-41)	41 (37-45)	45 (41-48)	51 (46-56)	
West	21 (19-23)	19 (15-22)	15 (13-18)	13 (10-17)	
Family size, No. (95% CI)	2.09 (2.05-2.14)	1.73 (1.66-1.80)	2.40 (2.32-2.47)	1.97 (1.86-2.08)	<.001
Family members with ASCVD, mean No. (95% CI)	1.08 (1.07-1.09)	1.07 (1.05-1.09)	1.10 (1.08-1.12)	1.12 (1.08-1.16)	.02
Cardiovascular disease risk factors					
Hypertension	78 (76-79)	81 (78-84)	78 (76-81)	81 (77-84)	.25
Diabetes	30 (28-31)	33 (29-36)	37 (34-39)	43 (38-48)	<.001
Dyslipidemia	74 (72-76)	73 (70-77)	74 (71-76)	71 (67-76)	.77
Current smoker^b^	13 (11-14)	12 (9-15)	25 (22-27)	18 (14-24)	<.001
Obesity^b^	33 (31-35)	33 (29-37)	42 (39-45)	39 (34-44)	<.001
Insufficient physical activity^c^	60 (58-61)	65 (61-68)	63 (61-67)	69 (64-73)	<.001
Medical comorbidities					
Arthritis	58 (56-60)	65 (61-68)	62 (60-65)	64 (59-70)	<.001
Cancer	28 (26-29)	31 (27-35)	23 (21-25)	24 (20-29)	<.001
Asthma	15 (14-16)	19 (15-22)	19 (17-22)	23 (19-28)	<.001
Chronic obstructive pulmonary disease	16 (15-17)	22 (19-26)	20 (18-22)	22 (17-26)	<.001
Hepatitis	0 (0-1)	0 (0-1)	0 (0-2)	0 (0-1)	.67
Chronic kidney disease	3 (2-4)	3 (2-5)	4 (3-6)	5 (2-9)	.14

Abbreviations: ASCVD, atherosclerotic cardiovascular disease; FPL, federal poverty level; GED, General Educational Development; NA, not applicable; OFH, objective financial hardship; SFH, subjective financial hardship.

^a^ Other race and ethnicity is defined as American Indian, Asian, or individuals of more than 1 race and ethnicity.

^b^ Obesity data are missing for 2017, and smoke data are missing for 2018.

^c^ Defined as less than moderate physical activity 5 times per week.

**Figure 1.**
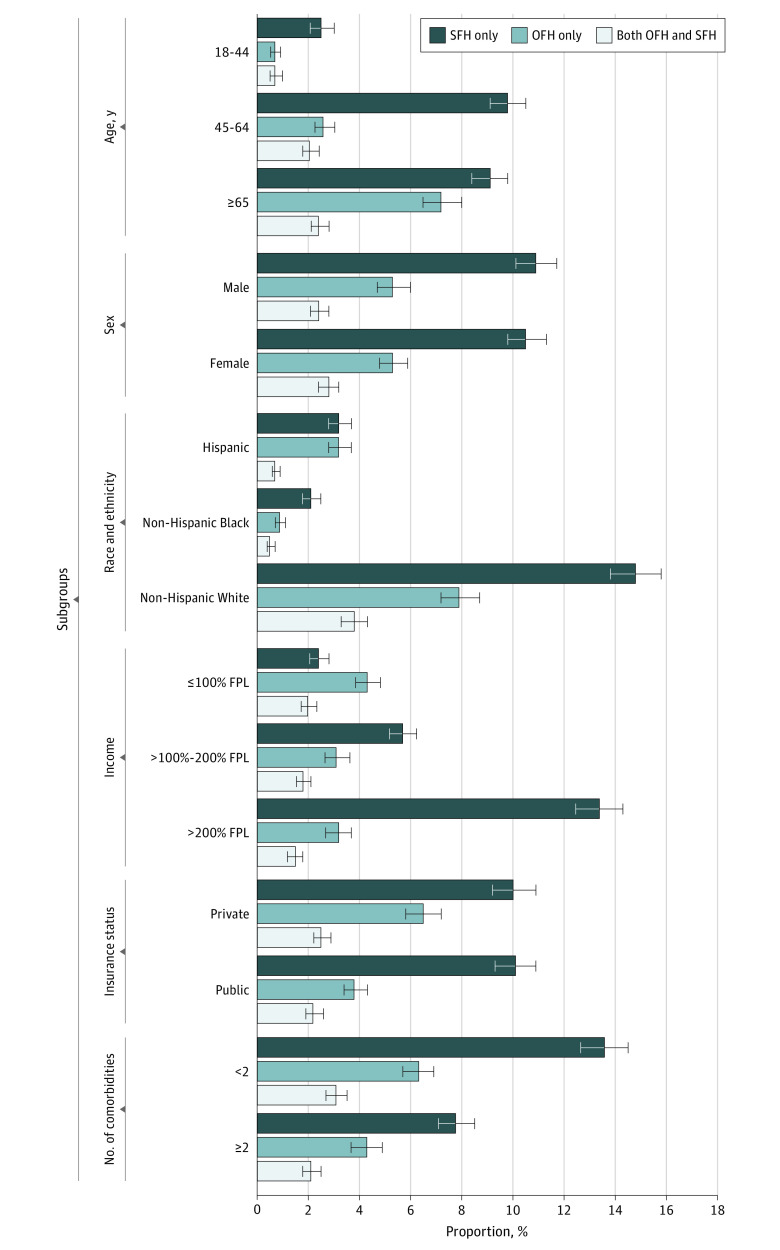
Proportion of Subjective Financial Hardship (SFH) and Objective Financial Hardship (OFH) in Families With a Member With Atherosclerotic Cardiovascular Disease (ASCVD), by Sociodemographic Subgroups FPL indicates federal poverty level; whiskers, 95% CI.

Across sociodemographic subgroups, subjective financial hardship was more common than objective financial hardship among families with a member with ASCVD. The highest absolute differences in unadjusted rates of subjective financial hardship vs objective financial hardship were observed in families in which index individuals were aged 45 to 64 years (10%; 95% CI, 9%-11% vs 3%; 95% CI, 3%-4%), in families with income greater than 200% of FPL (13%; 95% CI, 13%-14% vs 3%; 95% CI, 3%-4%), and among patients with public insurance (10%; 95% CI, 9%-11% vs 7%; 95% CI, 6%-7%) (Figure 1).

**Figure 2.**
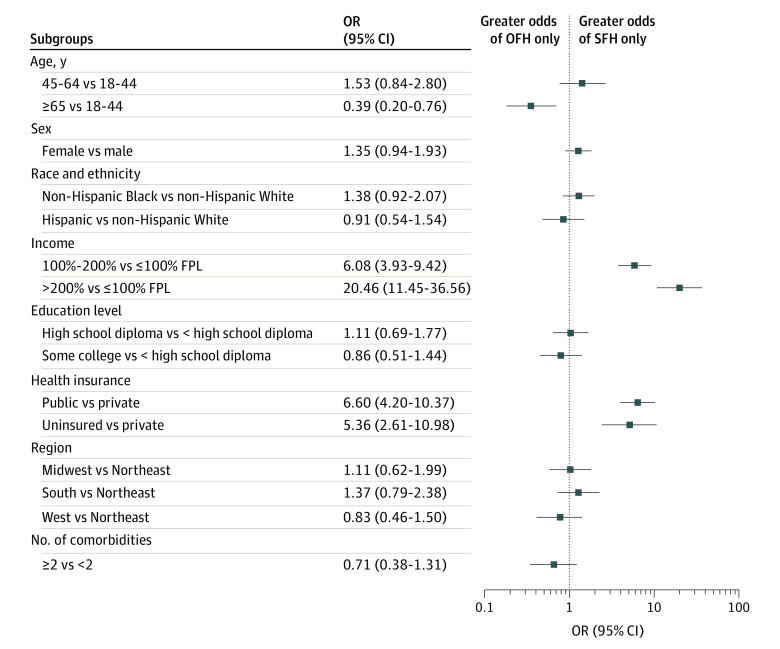
Odds of Subjective Financial Hardship (SFH) Only vs Objective Financial Hardship (OFH) Only in Families With a Member With Atherosclerotic Cardiovascular Disease (ASCVD), by Sociodemographic Subgroups FPL indicates federal poverty level.

In analyses that accounted for differences in patient and family characteristics, families in which the index participant with ASCVD was aged 65 years or older had significantly lower odds of subjective financial hardship than objective financial hardship (OR, 0.39; 95% CI, 0.20-0.76) compared with families in which the index person was between ages 18 and 44 years. Families with income greater than 200% of FPL had significantly higher odds of subjective financial hardship than objective financial hardship (OR, 20.46; 95% CI, 11.45-36.56) compared with families with income of 100% or less FPL, who more frequently had objective financial hardship from out-of-pocket health care spending. Furthermore, families with income of more than 100% to 200% of FPL had significantly higher odds of subjective financial hardship than objective financial hardship (OR, 6.08; 95% CI, 3.93-9.42). Individuals with public insurance or uninsured individuals had significantly higher odds of subjective financial hardship than objective financial hardship compared with individuals with private insurance (OR, 6.60; 95% CI, 4.20-10.37 for public insurance and OR, 5.36; 95% CI, 2.61-10.98 for uninsured) (Figure 2).

In addition, we found that uninsured status and public insurance status were associated with greater odds of both objective financial hardship and subjective financial hardship than only objective financial hardship compared with private insurance status (OR, 5.89; 95% CI, 2.61-13.26 for uninsured vs private insurance and OR, 2.11; 95% CI, 1.38-3.23 for public vs private insurance) (eFigure 1 in the Supplement). We also found that income higher than the FPL compared with less than or equal to the FPL was associated with a lower odds of co-occurrence of objective and subjective financial hardship than subjective hardship alone (eFigure 2 in the Supplement).

### Financial Hardship and Deferred or Forgone Care

Overall, 20% (95% CI, 19%-21%) of families reported deferred or forgone care, representing 1 or more members of the family delaying medical care, prescription medications, or dental care or forgoing necessary medical care, prescription medications, or dental care. When stratified by type of financial hardship, 9% had no financial hardship, 2% had objective financial hardship only, 7% had subjective financial hardship only, and 2% had both subjective and objective financial hardship. Families that had either objective financial hardship or subjective financial hardship and families with subjective financial hardship only had significantly higher risk-adjusted odds of deferred or forgone care compared with those without any financial hardship (OR, 2.42 [95% CI, 1.98-2.96] for either objective financial hardship or subjective financial hardship and OR, 2.76 [95% CI, 2.22-3.43] for subjective financial hardship only). Families with objective financial hardship alone did not have significantly different risk-adjusted odds of deferred or forgone care compared with those without financial hardship (OR, 1.03; 95% CI, 0.70-1.50). Families with subjective financial hardship alone had significantly higher risk-adjusted odds of self-reported deferred health care compared with families that reported objective financial hardship only (OR, 2.69; 95% CI, 1.79-4.06) (Table 2**)**.

**Table 2.** Odds of Deferred or Forgone Care Across Definitions of Financial Hardship

	Unadjusted OR (95% CI)	*P* value	Adjusted OR (95% CI)^**a**^	*P* value
Either SFH or OFH vs no financial hardship	2.45 (2.11-2.84)	<.001	2.42 (1.98-2.96)	<.001
SFH only vs no financial hardship	2.72 (2.31-3.21)	<.001	2.76 (2.22-3.43)	<.001
OFH only vs no financial hardship	1.21 (0.94-1.54)	.13	1.03 (0.70-1.50)	.89
SFH only vs OFH only	2.25 (1.74-2.92)	<.001	2.69 (1.79-4.06)	<.001

Abbreviations: OFH, objective financial hardship; SFH, subjective financial hardship.

^a^ Adjusted for age, sex, race and ethnicity, income, educational level, insurance status, region, 2 or more comorbidities, family size, and number of family members with atherosclerotic cardiovascular disease, hypertension, diabetes, dyslipidemia, current smoker, obesity, insufficient physical activity, arthritis, cancer, asthma, chronic obstructive pulmonary disease, hepatitis, and chronic kidney disease.

### Sensitivity Analyses

To further explore the general association between subjective financial hardship and objective financial hardship at a lower threshold for out-of-pocket health care spending, we conducted a sensitivity analysis with a 10% cutoff for the proportion of out-of-pocket costs to postsubsistence income that defined financial toxic effects. With this threshold, 46% of the included families endorsed either subjective financial hardship or objective financial hardship, 19% had objective financial hardship alone, 17% reported subjective financial hardship alone, and 10% had both. In sensitivity analysis that was restricted to 10 390 families (95% of the original) of patients with ASCVD with a postsubsistence income of more than $0, the differences in proportions of patients with objective financial hardship only and subjective financial hardship only by subgroups remained similar (eTable 2 in the Supplement).

## Discussion

Among families with 1 or more members with ASCVD in the US, 2 in 5 experienced health care–related financial hardship, but a focus on objective or subjective measures alone would have captured only about half of the burden and not identified those deferring health care. In this population, 1 in 10 families reported objective financial hardship only and 1 in 4 reported subjective financial hardship only. Across demographic and clinical subgroups, subjective financial hardship was more prevalent than objective financial hardship. A small minority of families reported both objective financial hardship and subjective financial hardship, highlighting the limitation of either measure in identifying the full scope of financial hardship in health care. Moreover, there was evidence that patients who reported subjective financial hardship were more likely to report forgoing or delaying health care compared with families that reported objective financial hardship, highlighting an important role of identifying both aspects of financial hardship for patients.

To our knowledge, this is the first study to highlight the nature of financial hardship in ASCVD across objective and subjective measures and the discordance across these seemingly aligned measures. We have previously found that approximately 14% of individuals with ASCVD reported significant objective financial hardship, but this finding could represent an underestimate when accounting for subjective financial hardship.^2^ Moreover, the limited overlap of both domains highlights that other studies that focus on subjective financial hardship alone have not captured the scope of the economic challenges.^1,9^ There have been previous reports of such a discordance in oncologic care, in which 16% to 73% of patients with cancer reported subjective financial hardship and 11% to 48% of patients reported objective financial hardship.^3,4^ However, even in these oncology studies, objective and subjective financial hardship were often not concurrently measured in the same sources.

This discrepancy between prevalence of subjective and objective financial hardship in the present study may be attributable to a few reasons. We found that families with subjective financial hardship only were more likely to forgo or delay medical care; hence, experiencing subjective financial hardship may lead to situations in which individuals may choose not to spend out of pocket on care (ie, avoiding health care in the first place), which would mean a lower prevalence of objective financial hardship. Although this phenomenon has not yet been reported in the cardiovascular disease literature, it has been described in the oncology literature; 2 studies published in 2013 reported significant and direct associations between subjective financial hardship and delayed or forgone care among patients with cancer.^16,17^ Furthermore, the results of the present study suggest that objective financial hardship, although commonly used as a measure of financial hardship, may be insufficient to show the true extent of financial burden, especially with respect to other expenses above and beyond out-of-pocket medical costs. Given that subjective financial hardship is much more prevalent than objective financial hardship (but there were also families with objective financial hardship only but no subjective financial hardship), lowering the threshold definition of objective financial hardship may identify more families with objectively defined financial hardship based on cost. Nevertheless, the present study suggests that a broader measure that combines both objective and subjective assessment of financial hardship is needed to fully assess the burden of financial hardship on patients.

The subgroup analyses suggest populations in which subjective financial hardship is significantly more likely than objective financial hardship and hence may be associated with more deferred or forgone care. We found that younger age (18-44 years), public health insurance status, and uninsured health insurance status were each associated with significantly greater odds of subjective financial hardship than objective financial hardship compared with age 65 years or older and private health insurance, respectively. These observations may be partially explained by the phenomenon of the deferral of cardiovascular disease medical services by uninsured or underinsured individuals until the onset of Medicare.^18^ Furthermore, younger individuals are more likely to have debt, including student loans and home mortgages,^19^ which may contribute to greater subjective financial hardship. The higher odds of subjective financial hardship than objective financial hardship among people who were uninsured compared with people with private insurance may also be owing to fewer visits to physicians among participants who are uninsured,^20^ which can be followed by greater deferral of care and less objective financial hardship. Targeted policy measures may need to be considered in these subgroups to mitigate financial hardship and the potential of deferral of medical care.

### Limitations

This study has limitations. First, MEPS is a cross-sectional study, and we were unable to longitudinally measure a family’s financial burden over time. Second, ASCVD diagnoses were based on self-report and may be affected by recall bias. Third, there is no criterion standard measure for subjective financial hardship or objective financial hardship. For objective financial hardship, there may be other unmeasured, large costs besides food expenses for families, and using a health care–related expense ratio relative to income of 20% as a surrogate measure for objective financial hardship may be an incomplete measurement (or underestimation) of a family’s overall financial burden. Furthermore, the specific definition of objective financial hardship used can have implications for prevalence of this outcome. However, similar measures have been used for objective financial hardship in previous studies of financial hardship,^1,2,9,21^ and using postsubsistence income to calculate objective financial hardship is a method of measurement endorsed by the World Health Organization.^22^ In the sensitivity analysis, we found that even when using a more conservative 10% threshold for the ratio of out-of-pocket expenses to income, most families still did not have subjective financial hardship and objective financial hardship concurrently. Fourth, individuals may have forgone or deferred care for reasons other than medical costs. However, regardless of the reason for deferral, we found this adverse health outcome to be overrepresented in the group reporting subjective financial hardship.

## Conclusions

In this cross-sectional, nationally representative study of US adults, 2 in 5 families of a patient with ASCVD experienced health care–related financial hardship, but a focus on objective or subjective measures alone would have captured only about half the burden and not identified those deferring health care. A comprehensive framework evaluating both objective and subjective measures is essential to monitor the financial consequences of health care.

## Notes

Supplement. **eTable 1.** *ICD-9* and *ICD-10* Codes for Risk Factors and Comorbidities **eTable 2.** Odds of SFH Only vs OFH Only in ASCVD, After Excluding If Postsubsistence Income Was Less than $0 **eFigure 1.** Odds of OFH Only vs Both OFH and SFH in Families With a Member With ASCVD, by Sociodemographic Subgroups **eFigure 2.** Odds of SFH Only vs Both OFH and SFH in Families With a Member With ASCVD, by Sociodemographic Subgroups
