# Supplementary material for: Measures of Financial Hardship From Health Care Expenses Among Families With a Member With Atherosclerotic Cardiovascular Disease in the US
Source: JAMA Health Forum. 2022 Jul 22;3(7):e221962. doi: 10.1001/jamahealthforum.2022.1962 (PMC9308060; doi:10.1001/jamahealthforum.2022.1962)
Supplement: Supplement. — eTable 1. ICD-9 and ICD-10 Codes for Risk Factors and Comorbidities eTable 2. Odds of SFH Only vs OFH Only in ASCVD, After Excluding If Postsubsistence Income Was Less than $0 eFigure 1. Odds of OFH Only vs Both OFH and SFH in Families With a Member With ASCVD, by Sociodemographic Subgroups eFigure 2. Odds of SFH Only vs Both OFH and SFH in Families With a Member With ASCVD, by Sociodemographic Subgroups [file jamahealthforum-e221962-s001.pdf]

# Supplementary Online Content

Wang SY, Valero-Elizondo J, Cainzos-Achirica M, Desai NR, Nasir K, Khera R. Measures of financial hardship from health care expenses among families with a member with atherosclerotic cardiovascular disease in the US. *JAMA Health Forum*. 2022;3(7):e221962. doi:10.1001/jamahealthforum.2022.1962

**eTable 1.** *ICD-9* and *ICD-10* Codes for Risk Factors and Comorbidities
**eTable 2.** Odds of SFH Only vs OFH Only in ASCVD, After Excluding If Postsubsistence Income Was Less than $0
**eFigure 1.** Odds of OFH Only vs Both OFH and SFH in Families With a Member With ASCVD, by Sociodemographic Subgroups
**eFigure 2.** Odds of SFH Only vs Both OFH and SFH in Families With a Member With ASCVD, by Sociodemographic Subgroups

This supplementary material has been provided by the authors to give readers additional information about their work.

© 2022 Wang SY et al. *JAMA Health Forum.*

**eTable 1.** ICD-9 and ICD-10 codes for risk factors and comorbidities

| Comorbidity | ICD-9 Codes or CCS codes | ICD-10 Codes |
|---|---|---|
| Hypertension | 401 | I10 |
| Diabetes | 250 | E11 |
| Dyslipidemia | 272 | E78 |
| Atherosclerotic Cardiovascular Disease | 410, 413, 414, 433-437, 440, 443 | I20, I21, I25, I63, G45, I70, I73, I79 |
| Arthritis | NA | M16, M17, M19 |
| Cancer | NA | C34, C53, C55, C56, C61, C64, C71, C76, C80, C85, C95 |
| Asthma | NA | J45 |
| Hepatitis | NA | B19 |
| Chronic Kidney Disease | NA | N18 |
| Chronic Obstructive Pulmonary disease | NA | J42, J43, J44 |

ICD, international classification of diseases

© 2022 Wang SY et al. *JAMA Health Forum.*

**eTable 2.** Odds of SFH only vs OFH only in ASCVD, after excluding if postsubsistence income was less than $0[a]

| | ASCVD | |
|---|---|---|
| | OR (95% CI) | P-value |
| Age | | |
| 18-44 | Ref | |
| 45-64 | 1.20 (0.66 – 2.19) | 0.54 |
| 65+ | 0.26 (0.14 – 0.50) | <0.001 |
| Sex | | |
| Male | Ref | |
| Female | 1.28 (0.85 – 1.93) | 0.24 |
| Race/ethnicity | | |
| White | Ref | |
| Black | 1.89 (1.17 – 3.06) | 0.01 |
| Hispanic | 1.09 (0.61 – 1.95) | 0.77 |
| Other | 1.06 (0.55 – 2.06) | 0.86 |
| Income Level | | |
| Poor | Ref | |
| Low | 1.86 (1.16 – 2.99) | 0.01 |
| Middle/High | 7.09 (3.84 – 13.12) | <0.001 |
| Education level | | |
| Less than High School | Ref | |
| High School/GED & Equivalent | 11.08 (0.63 – 1.83) | 0.78 |
| Some College or Higher | 0.75 (0.43 – 1.32) | 0.32 |
| Insurance Type, % (95% CI) | | |
| Private | Ref | |
| Public | 8.49 (5.26 – 13.71) | <0.001 |
| Uninsured | 7.08 (2.73 – 18.34) | <0.001 |
| Census Region, % (95% CI) | | |
| Northeast | Ref | |
| Midwest | 1.11 (0.59 – 2.09) | 0.75 |
| South | 1.40 (0.77 – 2.56) | 0.27 |
| West | 0.87 (0.45 – 1.67) | 0.68 |
| Comorbidities | | |
| <2 | Ref | |
| ≥2 | 0.75 (0.38 – 1.46) | 0.39 |

[a]Models adjusted for variables listed in table including obesity, insufficient exercise, dyslipidemia, hypertension, diabetes, smoking status, arthritis, cancer, asthma, chronic obstructive pulmonary disease, chronic kidney disease

© 2022 Wang SY et al. *JAMA Health Forum.*

**eFigure 1.** Odds of OFH only vs Both OFH and SFH in families with a member with ASCVD, by sociodemographic subgroups

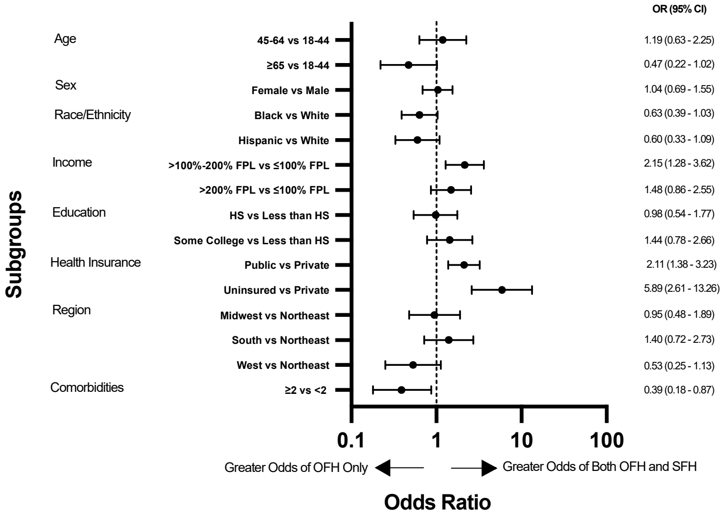


*Abbreviations*: ASCVD, atherosclerotic cardiovascular disease; HS, high school; NH, non-Hispanic; OFH, objective financial burden; SFH, subjective financial burden.

© 2022 Wang SY et al. *JAMA Health Forum.*

**eFigure 2.** Odds of SFH only vs Both OFH and SFH in families with a member with ASCVD, by sociodemographic subgroups

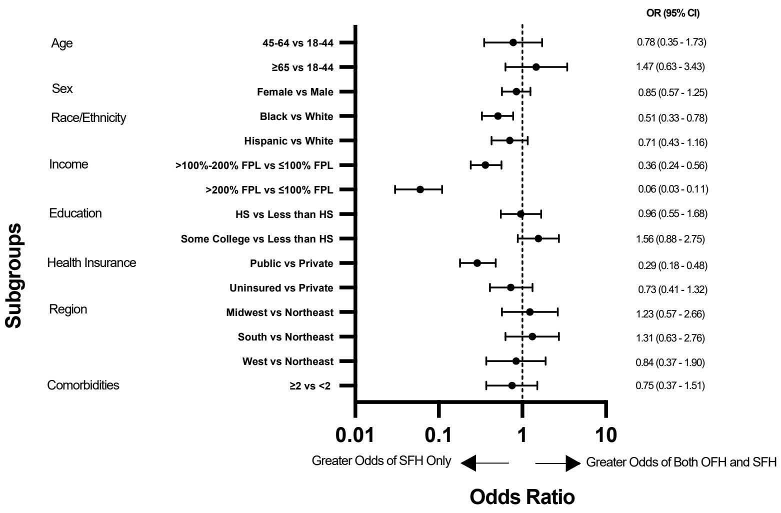


*Abbreviations*: ASCVD, atherosclerotic cardiovascular disease; HS, high school; NH, non-Hispanic; OFH, objective financial burden; SFH, subjective financial burden.

© 2022 Wang SY et al. *JAMA Health Forum.*
